# Supplementary material for: A UMLS-based spell checker for natural language processing in vaccine safety
Source: BMC Med Inform Decis Mak. 2007 Feb 12;7:3. doi: 10.1186/1472-6947-7-3 (PMC1805499; doi:10.1186/1472-6947-7-3)
Supplement: Additional file 13 — RAP application directory. Sets up directory for RAP (RDF application for PHP) source code [file 1472-6947-7-3-S13.gz › rap/doc/phpdoc/dataset/DatasetDb.html]

xml version="1.0" encoding="iso-8859-1"?


Docs For Class DatasetDb


## Class DatasetDb

Description

Description |
Methods (details)

Dataset implementation.

Superclass of datasetMem and datasetDb which contains shared functionality.

History:

- version: V0.9
- author: Daniel Westphal (http://www.d-westphal.de)
- author: Chris Bizer <chris@bizer.de>

Located in /dataset/DatasetDb.php (line 22)

```
Dataset
   |
   --DatasetDb
```

Method Summary

Description |
Methods (details)

DatasetDb
DatasetDb
(ADODBConnection &$dbConnection, DbStore &$dbStore, string $datasetName)

void
addAll
(Dataset $otherDataset)

void
addNamedGraph
(NamedGraphDb &$graph)

void
addQuad
(Quad &$quad)

void
clear
()

boolean
containsNamedGraph
(string $graphName)

boolean
containsQuad
(Resource $graphName, Resource $subject, Resource $predicate, Resource $object)

int
countGraphs
()

int
countQuads
()

NamedGraphDb
&createGraph
(string $graphName, [string $baseURI = null])

IteratorFindQuadsDb
&findInDefaultGraph
(Resource $subject, Resource $predicate, Resource $object)

IteratorFindQuadsDb
&findInNamedGraphs
(Resource $graphName, Resource $subject, Resource $predicate, Resource $object)

string
getDatasetName
()

NamedGraphDb
&getDefaultGraph
()

NamedGraphDb
&getNamedGraph
(string $graphName)

boolean
hasDefaultGraph
()

boolean
isEmpty
()

Array
listGraphNames
()

IteratorAllGraphsDb
&listNamedGraphs
()

void
removeNamedGraph
(string $graphName)

void
removeQuad
(Quad $quad)

void
setDatasetName
(string $datasetName)

void
setDefaultGraph
(DbModel &$graph)

Methods

Description |
Methods (details)

Constructor DatasetDb (line 60)

Constructor You can supply a Dataset name.

- access: public

DatasetDb

DatasetDb
(ADODBConnection &$dbConnection, DbStore &$dbStore, string $datasetName)

- ADODBConnection
  &$dbConnection
- DbStore
  &$dbStore
- string
  $datasetName

addAll (line 264)

Add all named graphs of the other dataset to this dataset

void

addAll
(Dataset $otherDataset)

- Dataset
  $otherDataset

addNamedGraph (line 119)

Adds a NamedGraph to the set.

void

addNamedGraph
(NamedGraphDb &$graph)

- NamedGraphDb
  &$graph

addQuad (line 285)

Adds a quad to the Dataset. The argument must not contain any wildcards. If the quad is already present, nothing happens. A new named graph will automatically be created if necessary.

void

addQuad
(Quad &$quad)

- Quad
  &$quad

clear (line 227)

Deletes all NamedGraphs from the set.

void

clear
()

containsNamedGraph (line 170)

Tells wether the Dataset contains a NamedGraph.

boolean

containsNamedGraph
(string $graphName)

- string
  $graphName

containsQuad (line 309)

Tells wether the Dataset contains a quad or quads matching a pattern.

boolean

containsQuad
(Resource $graphName, Resource $subject, Resource $predicate, Resource $object)

- Resource
  $graphName
- Resource
  $subject
- Resource
  $predicate
- Resource
  $object

countGraphs (line 236)

Returns the number of NamedGraphs in the set. Empty graphs are counted.

int

countGraphs
()

countQuads (line 351)

Counts the Quads in the RDF dataset. Identical Triples in different NamedGraphs are counted individually.

int

countQuads
()

createGraph (line 216)

Creates a new NamedGraph and adds it to the set. An existing graph with the same name will be replaced. But the old namedGraph remains in the database.

NamedGraphDb

&createGraph
(string $graphName, [string $baseURI = null])

- string
  $graphName
- string
  $baseURI

findInDefaultGraph (line 395)

Finds Statements that match a pattern in the default Graph. The argument may contain wildcards.

IteratorFindQuadsDb

&findInDefaultGraph
(Resource $subject, Resource $predicate, Resource $object)

- Resource
  $subject: or null
- Resource
  $predicate: or null
- Resource
  $object: or null

findInNamedGraphs (line 367)

Finds Statements that match a quad pattern. The argument may contain wildcards.

IteratorFindQuadsDb

&findInNamedGraphs
(Resource $graphName, Resource $subject, Resource $predicate, Resource $object)

- Resource
  $graphName: or null
- Resource
  $subject: or null
- Resource
  $predicate: or null
- Resource
  $object: or null

getDatasetName (line 110)

Return the Dataset name.

- access: public

string

getDatasetName
()

getDefaultGraph (line 140)

Returns a reference to the defaultGraph

NamedGraphDb

&getDefaultGraph
()

getNamedGraph (line 182)

Returns the NamedGraph with a specific name from the Dataset.

Changes to the graph will be reflected in the set.

- return: or null

NamedGraphDb

&getNamedGraph
(string $graphName)

- string
  $graphName

hasDefaultGraph (line 150)

Returns true, if an defaultGraph exists. False otherwise

boolean

hasDefaultGraph
()

isEmpty (line 255)

Tells wether the set contains any NamedGraphs.

boolean

isEmpty
()

listGraphNames (line 197)

Returns the names of the namedGraphs in this set as strings in an array.

Array

listGraphNames
()

listNamedGraphs (line 245)

Returns an iterator over all NamedGraphs in the set.

IteratorAllGraphsDb

&listNamedGraphs
()

removeNamedGraph (line 160)

Removes a NamedGraph from the set. Nothing happens if no graph with that name is contained in the set.

void

removeNamedGraph
(string $graphName)

- string
  $graphName

removeQuad (line 329)

Deletes a Quad from the RDF dataset.

void

removeQuad
(Quad $quad)

- Quad
  $quad

setDatasetName (line 92)

Sets the Dataset name. Return true on success, false otherwise

- access: public

void

setDatasetName
(string $datasetName)

- string
  $datasetName

setDefaultGraph (line 131)

Overwrites the existting default graph.

void

setDefaultGraph
(DbModel &$graph)

- DbModel
  &$graph

#### Inherited Methods


Inherited From Dataset

> Dataset::loadFromFile()  
> Dataset::loadFromString()  
> Dataset::serializeToFile()  
> Dataset::serializeToString()

Documentation generated on Thu, 7 Jul 2005 13:41:12 +0200 by phpDocumentor 1.3.0RC3
